# Physiological Responses Manifested by Some Conventional Stress Parameters and Biophoton Emission in Winter Wheat as a Consequence of Cereal Leaf Beetle Infestation

**DOI:** 10.3389/fpls.2022.839855

**Published:** 2022-07-06

**Authors:** Helga Lukács, Ildikó Jócsák, Katalin Somfalvi-Tóth, Sándor Keszthelyi

**Affiliations:** Institute of Agronomy, Hungarian University of Agriculture and Life Sciences, Kaposvár, Hungary

**Keywords:** biotic stress, delayed fluorescence, leaf damage, lipid oxidation, non-invasive imaging, *Oulema melanopus*, *Triticum aestivum*, ultra-weak photon emission

## Abstract

*Oulema melanopus* L. (*Coleoptera: Chrysomelidae*) is one of the most serious pests of winter wheat that causes peeling of the epidermis and tissue loss. The complex mapping of the physiological responses triggered by *O. melanopus* as a biotic stressor in winter wheat has not been fully explored with the help of non-invasive imaging and analytical assays, yet. The aim of the present work was to study the effect of *O. melanopus* on the physiological processes of winter wheat, especially on the extent of lipid peroxidation and antioxidant activity derived from tissue destruction, as well as photosynthetic ability. The results of the measurements enabled the identification of the antioxidant and lipid-oxidation–related physiological reactions, and they were reflected in the dynamics of non-invasive biophoton emissions. Our non-invasive approach pointed out that in the case of *O. melanopus* infestation the damage is manifested in tissue loss and the systemic signaling of the biotic stress may have reached other plant parts as well, which was confirmed by the results of antioxidant capacity measurements. These results indicate that the plant reacts to the biotic stress at a whole organizational level. We identified that the antioxidant and lipid-oxidation–related physiological reactions were reflected in the dynamics of two aspects of biophoton emission: delayed fluorescence and ultra-weak bioluminescence. Our research further supported that the non-invasive approach to stress assessment may complete and detail the traditional stress indicators.

## Introduction

The cereal leaf beetle, *Oulema melanopus* L. (*Coleoptera: Chrysomelidae*) is one of the most serious pests of cereals, especially winter wheat. The species is originally a Mediterranean fauna element, but it has been introduced in the whole Palearctic and Nearctic regions (Lesage et al., 2007). The presence and damages of this pest are very significant due to the extended distributions of it and its host plants, which is confirmed by in-crop pesticide treatments against it by means of some residual (Tanaskovic et al., 2012) and other biological in-crop applications (Mazurkiewicz et al., 2021).

The typical symptoms of both adults and larvae on the host plant caused by cereal leaf beetle are thin and long lines, where initially the epidermis of the leaf has been peeled. Under uncontrolled circumstances, these damages can aggravate in several cases even when the majority part of photosynthetic surfaces could also impair (Bieńkowski, 2010). A field of cereals looks weathered and chloritized, but is never completely destroyed. First and second leaves of plants are mostly inhabited by larvae (Groll and Wetzel, 1984), where damage is usually no more than 40% of the total (Philips et al., 2011). In case the leaves of cereals are damaged by some biotic or abiotic factors, they generally lag behind in their development and have a decreased nutrient integration, occurred water management disturbance which eventually leads to quantitative and qualitative yield loss (Steinger et al., 2020).

The *in vivo* and non-invasive approach in plant stress detection has been considered to be of high importance, and the biophoton emission measurement is one of these techniques whose application has gained attention in the last two decades (Bodemer et al., 2000; Birtic et al., 2011; Kobayashi et al., 2014; Rui-Rui et al., 2015; Kamal and Komatsu, 2016; Kan et al., 2017; Prasad et al., 2017; Oros and Alves, 2018; Zhou et al., 2019; Jócsák et al., 2020; Chen et al., 2021; Pónya et al., 2021). Plants emit photons under certain circumstances, such as energy release from the photosynthetic apparatus of the non-utilized photons in the electron transport chain of photosystem II (PSII). This phenomenon is the delayed fluorescence (DF) that reflects the integrity state of the photosynthetic apparatus. DF only occurs in photosynthetic tissues with decay times from milliseconds to minutes (Bodemer et al., 2000). At this point, some of the electrons in the photosynthetic electron transport chain flow back to the reaction center, where the chlorophyll molecules reach an excited state and emit photons, such as red luminescence. Research revealed that DF decay kinetics are suitable for the *in vivo* determination of the homeostatic state of plants, nevertheless the extent of DF and its decay dynamics are suitable for stress assessment purposes (Goltsev et al., 2009, Sánchez-Moreiras et al., 2020). Bodemer et al. (2000) found that DF decay differs in 3-(3,4-dichlorophenyl)-1,1-dimethylurea (DCMU)-poisoned *Chlorella* spp. culture compared with healthy culture. Zhou et al. (2019) used DF to assess the effects of drought stress and salinity on barley, but to our knowledge, not for biotic stressors.

Another source of photon emission is related to the mitochondrial processes, the electron transport chain which emits photons through the relaxation of its excited components. Furthermore, the oxidation of lipids (Zhou et al., 2019) produces photon-emitting reactive oxygen species (ROS), such as triplet carbonyls and singlet oxygen, as well as reactive nitrogen species (RNS) (Bennett et al., 2005), the detection and visualization of which is possible with a highly sensitive charge-coupled device (CCD) (Jócsák et al., 2020).

These processes can be utilized in stress-related researches in plants since the strength of the signals of both DF and ultra-weak bioluminescence (UWLE) differ under the metabolic and tissue structure alterations triggered by stressors of either abiotic. UWLE was formerly used for the characterization of heat stress (Kobayashi et al., 2014), flood (Kamal and Komatsu, 2016), osmotic stress (Rui-Rui et al., 2015), or cadmium (Jócsák et al., 2020), similarly to biotic stressors, such as leaf wound in *Spathiphyllum* (Oros and Alves, 2018) and *Arabidopsis* (Prasad et al., 2017), or the infestation of two spotted-spider mite (*Tetranychus urticae* L.) (Pónya et al., 2021). These investigations commonly utilize the possibility to differentiate among the effects of stressors *via* imaging and parameterizing of the bioluminescence measurements, but to this date there were no studies on the tissue alterations of *O. melanopus* on winter wheat.

Several studies carried out research on the effects of leaf impairment triggered by various factors on reproductive traits (Macedo et al., 2007; Shao et al., 2010; Steinger et al., 2020), nutrient content (Buráňová et al., 2015; Hamnér et al., 2017), and adaptive ability (Chen et al., 2016) of winter wheat. Nevertheless, the complex mapping of the physiological responses triggered by this biotic stressor in winter wheat has not been fully explored with the help of non-invasive imaging and analytical assays, yet. In this context, the aim of the present work was to study the effect of *O. melanopus* on the physiological processes of winter wheat, especially on the extent of lipid peroxidation and antioxidant activity derived from tissue destruction, as well as photosynthetic ability. In addition, we were also aimed to identify whether these physiological reactions are reflected in the dynamics of the non-invasive measurement, imaging, and analysis of biophoton emission.

## Materials and Methods

### Sampling and Experimental Setting

To determine the physiological response of winter wheat caused by *O. melanopus*, mixed-gender adults were collected from an insecticide-free environment. The collection was carried out in early April 2021 in the Zselicszentpál area (Hungary, Somogy county, GPS coordinates: 46°30′84′′N, 17°82′08′′E). Adults were collected using an entomological sweeping net. The temperature range during the rearing of insects in the climate chamber was 20 ± 1°C. Relative humidity was maintained at 60 ± 5%, and photoperiodic setting was 18L:6D, corresponding to the insect vitality optimum (Mazurkiewicz et al., 2019).

In parallel, healthy, damage-free winter wheat seeds were sown in 19-cm diameter plastic pots (80 seeds/pot), which were placed in a Pol-Eco Apartura KK 1450 climate chamber (POL-EKO-APARATURA sp.j. ul. Kokoszycka172C 44–300 Wodzisław Śląski, Poland) at 20°C, 120 μM m^–2^ s^–1^ light intensity for 16 h as daylight conditions and 16°C; 0 μM m^–2^ s^–1^ light intensity for 8 h as night conditions. When seedlings reached the 2–3 leaved stage, 5 pots continued to grow without changing their conditions, and 5 pots were treated with 20 images of the model beetles (*O. melanopus*) per pot under the isolator covered with well-ventilated textiles. Subsequently, after leaf damage, stress analytical evaluation and non-invasive imaging were taken on the sixth day of insect application to determine the extent of oxidative stress and its visual display. First, the non-invasive type of the measurements were conducted starting with ultra-weak photon emission (UPE). After the completion of the UPE measurement, the soil plant analysis development (SPAD) measurement was done. The sequential order of the measurement was chosen to avoid any potential stress on the plants including even the light pressure that the usage of SPAD equipment poses on the leaf blades of the seedlings. Subsequently, the seedlings were sampled for the fresh/dry weight (DW) determination, ferric reducing antioxidant power (FRAP), and malondialdehyde (MDA) measurements when the whole above-ground part of the plants was used. The sampling method was the following: the above-ground part of the seedlings was cut into ∼0.5-cm pieces. After that the pieces were mixed thoroughly to create an average sample for the fresh DW and for antioxidant capacity and lipid peroxidation measurement. The measurements were repeated three times per pot and the chemical analyses were measured in three parallels per each pot.

### Tissue Damage Determination

**FIGURE 1:**
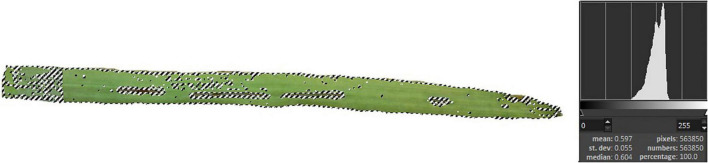
Leaf surface destruction caused by *O. melanopus*. Five leaves were scanned and the pixels of damaged leaves were determined in the percentage of reduction of the photosynthesizing surface.

Similar to other leaf analysis-based experiments (Sorgini et al., 2019; Zhang et al., 2019; Wang et al., 2020), damaged leaves were scanned to objectively determine leaf surface destruction. Five leaves were scanned and the number of pixels per each image were determined and the pixel number of the white background was subtracted, resulting in the pixel number of the whole leaf. Then, pixel points of differently colored areas were designated using the GIMP 2.10.8 software, and thus the pixels of damaged leaves were determined in the percentage of reduction of the photosynthesizing surface (Figure 1).

### Determination of Fresh/Dry Weight Ratio

A total of 1 g of leaves were measured by an Ohaus Discovery DV215CDM (Ohaus Corporation 1.800.672.7722 7 Campus Drive, Suite 310 Parsippany, NJ 07054, United States) analytical scale weighing up to 5 decimal places. Subsequently, leaves were dried in a drying cabinet (Memmert SLE 600, Memmert GmbH + Co. KG, Aeussere Rittersbacher Strasse 38, D-91126 Schwabach, Germany) at 60°C for 24 h. Then the weight of the dried samples was recorded. Fresh/DW ratio was expressed as % and it was calculated by [(wx − w0)/wx] × 100, where the wx is the DW and w0 is the fresh weight.

### Measurement of Lipid Peroxidation

Malondialdehyde content was determined by the thiobarbituric acid (TBA) reaction with some modifications to the original method of Heath and Packer (1968). Samples of 0.5 g were homogenized with 2 mL of 0.1% trichloroacetic acid (TCA) in cold mortars from which 1.8 mL was transferred to 2 mL microtubes with automatic pipettes. To this solution, 40 μL of 20% butylated hydroxytoluene (BHT) in absolute ethanol was added to stop further lipid oxidation. The solutions were vortexed for 15 s and centrifuged at 18,600 × *g* for 10 min at 4°C. From the clear supernatant, 0.25 mL was added to 1 mL of 20% TCA containing 0.5% TBA, gently mixed, and briefly centrifuged for 5 s. The solutions were incubated in a water bath (Julabo ED-5M) for 30 min at 96°C. The reactions were stopped by cooling the solutions immediately on ice followed by centrifugation at 10,000 rpm for 5 min. Absorbance at 532 and 600 nm was recorded using a SmartSpec™ Plus spectrophotometer, and MDA concentration was calculated by subtracting the non-specific absorption at 600 nm from the absorption at 532 nm using an absorbance coefficient of extinction, 156 mM^–1^ cm^–1^. The results were expressed as nmol g^–1^ in DW.

### Ferric Reducing Antioxidant Power Assay

Total antioxidant activity is measured by the modified assay of FRAP of Benzie and Strain (1999). The constituents of the FRAP reagent were the following: acetate buffer (300 mM pH 3.6), 2,4,6-tripyridyl-*s*-triazine (TPTZ) 10 mM in 40 mM HCl, and FeCl_3_6H_2_O (20 mM). The working FRAP reagent was prepared by mixing acetate buffer, TPTZ, and FeCl_3_6H_2_O in the ratio of 10:1:1 at the time of use. The standard solution was 10 mM ascorbic acid (AA) prepared freshly at the time of measurement. To 0.1 mL of the supernatant was added 2.9 mL of FRAP reagent in 5-mL screw cap centrifuge tubes, vortexed in a 37°C water bath (Julabo ED-5M, JULABO GmbH 77960 Seelbach/Germany) for 4 min, and the absorbance was measured at 593 nm against a blank with a BIORAD SmartSpec™ Plus spectrophotometer (Bio-Rad Ltd., 1000 Alfred Nobel Drive Hercules, CA 94547, United States). The FRAP values of the samples were determined were of AA equivalent (mmol AA equivalent g^–1^ DW) based on the AA calibration curve, as the averages of three independent measurements (Benzie and Strain, 1999; Szõllõsi and Szõllõsi Varga, 2002).

### Determination of Relative Chlorophyll Content

The SPAD index, a non-invasive measurement for relative chlorophyll content, was measured by reading 10 individual points on 10 seedlings of each treatment with SPAD (Soil Plant Analysis Development–SPAD-502; Konica Minolta Sensing Inc., Japan) equipment. In a wheat leaf with ∼10–15 cm long, measurements were taken along the full length of each leaves approximately every 1–1.5 cm.

### Non-invasive Imaging of Stress Reactions in Plant: Delayed Fluorescence and Ultra-Weak Photon Emission

For measuring UPE, the NightShade LB 985 In Vivo Plant Imaging System (Berthold Technologies GmbH & Co. KG, 75323 Bad Wildbad, Germany) equipped with a sensitive, thermoelectrically cooled slow-scan NighOwlcam CCD device has been employed. The instrument was controlled by the IndiGo™ 2.0.5.0. software. Intensities of light were converted into counts per second (cps) by using the controlling software. The exposure time was kept at 60 s using a pixel binning of 2 × 2. During the duration of taking the images both the “background correction” and the “cosmic suppression” options were enabled to ensure the elimination of high-intensity pixels potentially caused by cosmic radiation. One pot for each treatment of the seedlings to be imaged was placed into the dark imaging box. In the first part of the measurement, DF signal was measured immediately after placing the pots in the dark chamber for 10 min. Thereafter, the samples were continued to be kept in dark to provide sufficient time for dark adaptation, and from the 30th minute, for the pots spent in the dark, bioluminescence data was also acquired for 10 min.

### Statistical Analysis

One-way ANOVA was assumed to applied to determine the differences between samples. To do so, two conditions must be fulfilled. Firstly, the samples must be normally distributed, secondly the samples must have homoscedasticity. Shapiro–Wilks test was used to determine the distribution. The null hypothesis was that the sample is normally distributed, which was accepted when the *p*-value exceeded 0.05. Depending on the distribution, i.e., the sample is normally or not-normally distributed, two methods were applied to calculate the homogeneity of variances: Bartlett test for normal distributions and Flinger–Killeen non-parametric test for non-normal distributions. When the distribution of the two samples was different, the less robust Flinger–Killeen test was used to determine homoscedasticity. Since the results of testing conditions supported the application of one-way ANOVA only in the case of fresh/DW data, Wilcoxon-test was used to prove the differences between samples for the rest of the measured parameters. When both samples (control + infested) have the same distribution, the type of homoscedasticity test is unequivocal. In the case of MDA, the Flinger–Killeen test was chosen with higher reliability for determining the homogeneity of variance.

### Statistical Outputs of Homogenous Distribution and Significant Differences of the Examined Stress Indices

Table 1 presents the results of the statistical analysis of the measured physiological parameters, such as relative chlorophyll content, antioxidant capacity, and lipid oxidation; and biophoton emission-related parameters: DF and UPE.

**TABLE 1:** Statistical analysis of the examined stress parameters in damaged wheat caused by *O. melanopus*: ANOVA analysis of fresh/dry weight ratio and Wilcoxon tests of relative chlorophyll content (SPAD), antioxidant capacity (FRAP), lipid oxidation (MDA), delayed fluorescence (DF), and ultra-weak photon emission (UPE).

Summary	Results of ANOVA for fresh/dry weight ratio					
	
Groups	Count		Sum		Average	Variance
Control	5		45.318		9.063	0.017
*O. melanopus* infested	5		60.354		12.071	0.106

**Source of variation**	**SS**	**df**	**MS**	***F***	***p*-value**	***F* critical value**

Between groups	22.608	1	22.608	384.891	5.8 × 10^–8^	5.317
Within groups	0.495	8	0.061			
Total	23.104	9				

	***p*-value**	**Results of Wilcoxon test**				

MDA	1.478 × 10^–7^	There are significant differences between groups				
FRAP	3.383 × 10^–6^	
SPAD	2.200 × 10^–16^	
UPE	2.200 × 10^–17^	
DF	1.520 × 10^–3^	

The results of the Shaphiro–Wilks test indicated that UPE and MDA values showed normal distribution but the test of the parameters (SPAD, FRAP, and DF) did not. Furthermore since the variance of the samples was so pronounced, the ANOVA was only applicable for the statistical analysis of the fresh/DW ratio values. According to the results for one-way ANOVA and Wilcoxon tests, all parameters showed significant differences (Table 1; more detailed results in Supplementary Material 1).

## Results

### Changes in the Photosynthetic Tissue and Relative Chlorophyll Content

**FIGURE 2:**
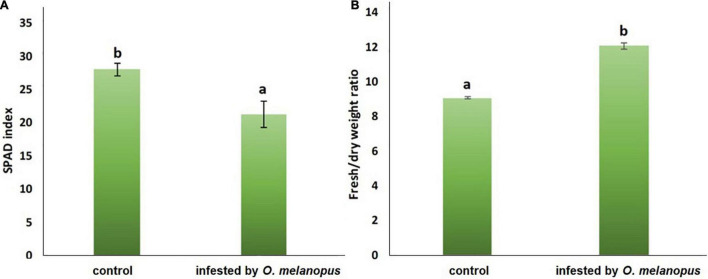
**(A)** Relative chlorophyll content (SPAD index; *n* = 10) and **(B)** fresh: dry weight ratio (*n* = 10) of healthy and *O. melanopus* infested wheat leaves. Mean values and standard deviations are presented. Letters represent significant differences within the treatments.

The damage of *O. melanopus* showed serious tissue damage during the 6 days of infestation. Mean of leaf surface destruction caused by *O. melanopus* was 12.280 ± 1.323%, the average of five investigated leaves (Figure 1). The results of the relative chlorophyll content (SPAD index) and fresh/DW ratio are presented in Figure 2. The infestation of *O. melanopus* manifested as a significant decrease in the SPAD index from 28.2 to 20.6. The Shapiro–Wilks normality test showed that the SPAD data did not show normal distribution (*p* > 0.05), and thus was analyzed with the Wilcoxon test (Table 1) and revealed a significant (*p* = 2.2 × 10^–16^) 27% decrease in total (Figure 2). The normality test resulted in normal distribution for fresh/dry mass ratio, and consequently the one-way ANOVA was used and proved a significant (*p* = 5.84 × 10^–16^) increase of *O. melanopus* infested wheat: 24.9%, from 9.064 to 12.071.

### Changes in Antioxidant Capacity and Lipid Oxidation

**FIGURE 3:**
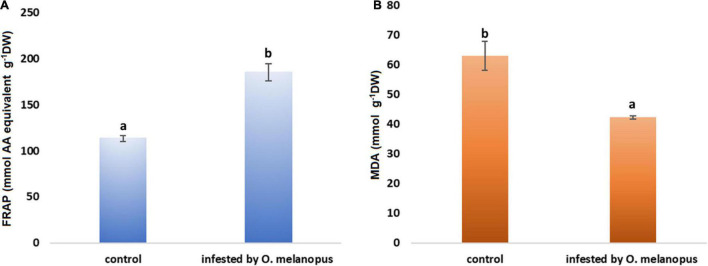
Effect of *O. melanopus* infestation on **(A)** antioxidant capacity (*n* = 15) (mmol AA equivalent g^– 1^ DW) and **(B)** lipid oxidation (*n* = 30) (m g^– 1^ DW MDA equivalent) in wheat leaves. Mean values and standard deviations are presented. Different letters represent significant differences within the treatments.

The FRAP values reflect that part of the overall antioxidant state of plant tissues that is possible to determine with the method of ferric reducing antioxidant capacity. The Shapiro–Wilks normality test resulted that the antioxidant capacity data did not show normal distribution (*p* > 0.05), and the Wilcoxon test (Table 1) was used which showed that the increase was significant (*p* = 3.383 × 10^–6^) in the FRAP values of the *O. melanopus*-infested wheat samples (11.43 μg AA equivalent g^–1^ FW) compared with the healthy leaves (6.92 μg AA equivalent g^–1^ FW; Figure 3).

Lipid oxidation is a stress indicator of membrane structure- and function-related processes, such as the damage caused by *O. melanopus*. The results of MDA level measurement showed the decrease in MDA content in the tissues of *O. melanopus*-infested wheat. The Shapiro–Wilks normality test resulted that the lipid oxidation data did not show normal distribution (*p* > 0.05), thus the data were analyzed with the Wilcoxon test (Table 1), which proved that the decrease in MDA content was significant (*p* = 1.478 × 10^–7^). The control MDA level was 16.91 nM g^–1^ FW and the *O. melanopus*-infested level dropped to 14.87 nM g^–1^ FW, i.e., 13%, which is a statistically significant (*p* ≤ 0.05) decrease induced by the biotic stressor: *O. melanopus*.

### Changes in the Sum of Overall Count per Second Values of Delayed Fluorescence and Lipid Oxidation Related Bioluminescence

**FIGURE 4:**
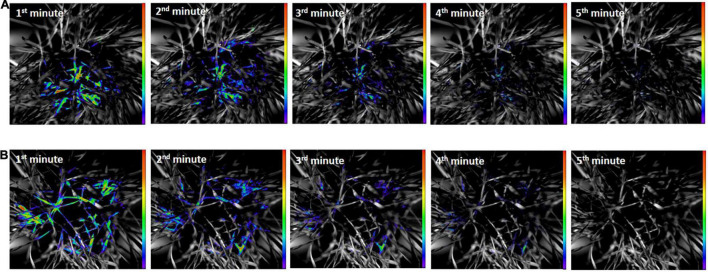
**(A)** Delayed fluorescence decay of healthy and **(B)** *O. melanopus* infested leaves during the first 5 min of the measurement. The intensity of color bar shows signal intensity detected by the equipment and converted into a scale of color intensity *via* IndiGo™ 2.0.5.0. software.

The results of DF decay of healthy and *O. melanopus* infested leaves during the first 5 min of the measurement are presented in Figure 4. The images reveal a distinct difference between the healthy and stressed samples. The DF signal of the healthy plants was highly intensive during the first minute of the measurement in both sample types, which was depicted as intensive orange and red pseudocolors in the images. A decreasing tendency of signals was obtained from the second minute of the measurement in heathy plants; however, the photon emission signals arising from the *O. melanopus* infested leaves were lower as it was indicated by the pseudocolors. This tendency continued during the fourth minute and the photon emission reached low intensity by the end of the fifth minute.

**FIGURE 5:**
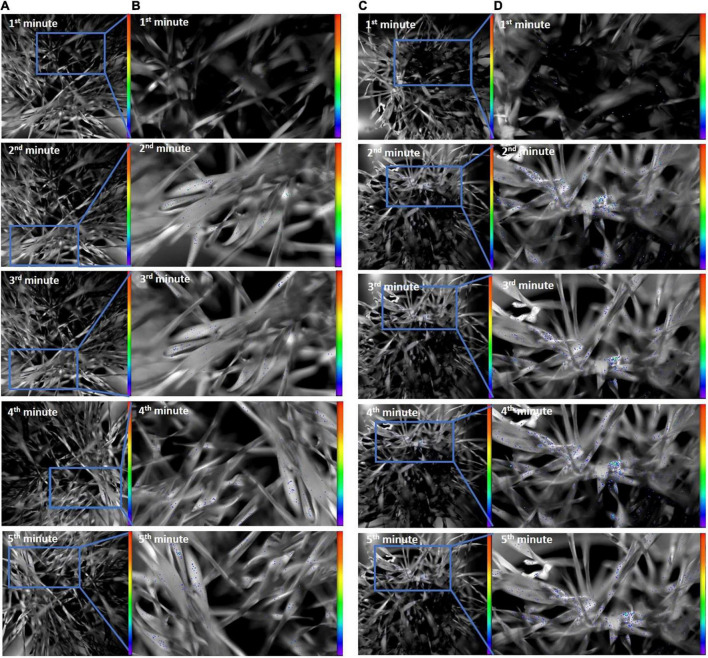
Ultra-weak photon emission of (**A**: original; **B**: enlarged) healthy and (**C:** original; **D:** enlarged) *O. melanopus*-infested leaves during the first 5 min of measurement. The intensity of color bar shows signal intensity detected by the equipment and converted into a scale of color intensity *via* IndiGo™ 2.0.5.0. software.

Figure 5 shows the results of UPE both in the original size and due to UPE in an enlarged version of typical areas with considerable UPE signals. The images of UPE signals showed differences between the healthy and *O. melanopus*-infested samples, and according to the pseudocolor intensity, it indicated an increase of stressed plants in all the first 5 min of the measurement compared to that of the healthy samples.

**FIGURE 6:**
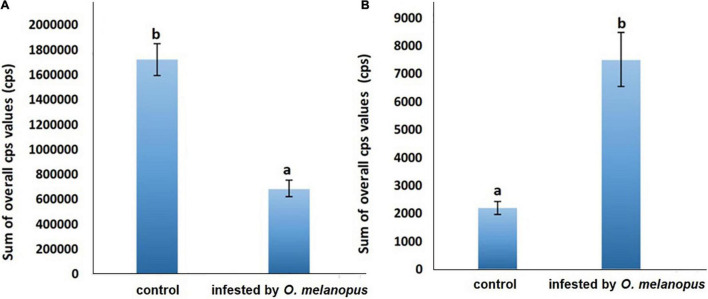
Sum of overall count per second **(A)** DF and **(B)** UPE values of control and *O. melanopus*-infested wheat. Mean values (*n* = 3) and standard deviations are presented. Letters represent significant differences within the treatments.

Besides the acquisition of images, the fluorescence data were also analyzed to derive objective parameters for a precise evaluation of the putative trends and tendencies depicted by the images. DF measurements were followed consecutively by UWLE data acquisition after a completed dark adaptation period of 30 min (Figure 6). This measurement setup enabled a distinction between photosynthetic- and lipid-oxidation-related processes. The Shapiro–Wilks normality test resulted that the both DF and UPE data did not show normal distribution (*p* > 0.05), and thus was analyzed with Wilcoxon test.

The presented data are the summary of all photon counts during 10 min of data acquisition for both DF and UPE (Figure 6). The results show an opposite trend of the changes in DF and UPE indicated before in the presented images (Figures 4, 5), but both types of measurements resulted in a significant difference in the sum of overall photon count per second values. However, in the case of DF, the infestation of *O. melanopus* resulted in a 250% significant decrease (*p* = 0.001152) compared to the control plants from 1.72 × 10^6^ to 6.88 × 10^5^. On the contrary, the direction of the changes of UPE was opposite to that of the DF values and resulted in a significant (*p* = 2.2 × 10^–17^) increase (Table 1). UPE was more than 300% higher than in the case of the control from 7499.688 to 2197.424 (Figure 6).

### Changes of Overall Count per Second Values of Delayed Fluorescence and Lipid Oxidation-Related Bioluminescence

To identify the temporal dynamics of *O. melanopus*-induced changes in photon emission, the time-course analysis of the detected photon signals was also conducted besides the evaluation of the sum of the overall cps values. Therefore, both DF and the consequent UPE were measured for 10 min.

**FIGURE 7:**
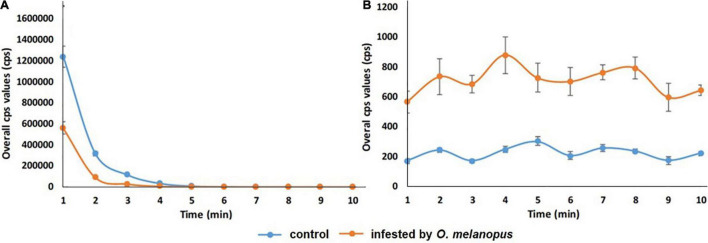
Temporal changes of overall count per second **(A)** DF and **(B)** UPE values of control and *O. melanopus*-infested wheat during a 10-min measurement period. Mean values (*n* = 5) and ±SD are presented.

The results show a distinctive trend in both the phenomena. DF has a highly sharp decreasing tendency in first few minutes of the measurement, and from the fifth minute the data decays toward zero, but does not reach it. After dark adaptation was completed, the UPE values reached a quasi-steady state that significantly differed according to the sample types for the whole duration of our observations (Figure 7).

## Discussion

The indication of this research was to visualize and gain parameters of the physiological effects regarding key metabolic pathways, such as photosynthesis and lipid oxidation affected by a biotic stressor, *O. melanopus*. Firstly, the tissue structure disruption was determined and analyzed by pixel distinction, and the damage was objectively determined as it was done several times before (Bieńkowski, 2010; Philips et al., 2011). After 6 days of *O. melanopus* infestation, there was tissue loss that was manifested in the decrease of chlorophyll content, which was indicated by the SPAD index and increased fresh/dry ratio values. The phenomenon of tissue loss is a typical symptom of *O. melanopus* infestation and it has been documented that the extent of the loss of photosynthetic tissue could be so pronounced that photosynthetic efficiency drops (Olfert et al., 2004). The results of relative chlorophyll content and fresh/DW ratios indicate mechanisms of altered photosynthetic system and tissue integrity.

The analytical investigations of antioxidant capacity and lipid oxidation, along with the measurements of DF and UPE, provided a deeper insight not only into the underlying mechanisms, but in the case of the two latter to the temporal dynamics as well. The dynamics of DF and UPE were opposite in their trends and the scale of the emitted photons were distinct. These changes and differences originated from the distinct nature of the underlying processes.

In the initial phase of photon emission measurement right after the placement of the plants into the dark chamber, the processes of the photosynthesis started to gradually cease, resulting in DF. It is well-known that dark-adaptation-related phenomenon of photosynthesizing tissues, the decay of which takes more time, if the plant is in an intact and healthy condition. However, DF ceases more rapidly if the plant is under the effect of a stressor of any kind, in our case a biotic one: *O. melanopus*. DF decay kinetics were proved to be proper tools for stress assessment purposes, mostly in the case of abiotic stressors (Bodemer et al., 2000; Kan et al., 2017; Zhou et al., 2019; Chen et al., 2021), and according to our results, for a biotic stressor as well. In accordance to the findings of Hennecke and Brüx (2012), our results also point to a relationship between UPE and the oxidation state. These authors showed that a healthy plant emits DF considerably more intensively, than a stressed one as opposed to the phenomenon of UPE; when the photon-emission is a sign of the presence of stressors, and thus stressed plants emit photons more intensively than non-stressed ones (Hennecke and Brüx, 2012; Jócsák et al., 2020).

The results of tissue loss quantification showed the destructive effect of *O. melanopus* infestation, which obviously included the loss of membrane system as well, that consequently reflected as a decrease in the colorimetric oxidative stress assessment assay. That is why according to our results, MDA content, as an indicator of lipid oxidation processes, did not completely prove to be suitable for the proper characterization of the degree of stress induced by *O. melanopus*. This phenomenon can be explained by the type of damage because the investigated impairment was triggered by a pest, which typically causes direct destruction of plant tissues. As opposed to some hiding lifestyle, arthropod pests, such as cereal leaf miner (*Agromyza nigrociliata* Hendel, 1931), which trigger an indirect covertly damage which will not occurred on the host plant (not directly affecting the examined photosynthetic tissues, Roik and Walczak, 2012). The reason for this may lie in the fact that the chewing action leads to tissue loss, which may interfere with the results of the analytical measurement. The formation of TBA reactive substances that are possible to detect *via* the formation of malondialdehyde are mostly linked to membrane lipids, the degradation of which leads to an accelerating reaction of oxidized forms of lipid membranes. In the case of *O. melanopus*, however, it was not possible to properly quantify as a consequence of tissue loss.

Nevertheless, the results of UPE enlightened this problem from a different angle. Until recently, the recognition of ROS as detrimental products of plant metabolism has been toned since it was confirmed that ROS act as signaling molecules with the capability of mediating environmental signals toward the genetic functionality of the cells leading to stress-responsive alteration of gene expression (Sewelam et al., 2016). These environmental signals include both abiotic stressors and biotic ones, such as pathogens, herbivores, and wounding, as we have experienced during our research work, since ROS are curial components of host defense responses leading to the increased synthesis of antioxidants (Bhatia et al., 2004), as indicated in our research work by the increased FRAP values. Different kinds of ROS, such as hydrogen peroxide, superoxide, or singlet oxygen combined with their production sites, such as plastids, cytosol, peroxisome, or apoplast, trigger distinctive physiological and molecular responses (Gadjev et al., 2006). One way of fulfilling these signaling roles is that ROS such as H_2_O_2_ is able to diffuse through cells *via* aquaporin (Borisova et al., 2012), leading to systemic responses (Sewelam et al., 2016). Although in the case of *O. melanopus* infestation, the damage is manifested in tissue loss, the systemic signaling of the biotic stress may reach other plant parts as well, which led to increased UPE signals. The results of antioxidant capacity measurements further strengthen this idea, since, despite the tissue loss, the non-enzymatic ferric reducing antioxidant capacity significantly increased, indicating that the plant reacts to the biotic stress on a whole organizational level.

Based on the present results, the changes of the healthy and *O. melanopus* infested wheat showed that the insect causes severe damage to wheat that was visualized (Figure 4) and parametrized (Figure 5) through the detection of photon signals emitted by the plants. Furthermore, our results indicate the possibility to capture the signaling mechanisms of infestation, since despite the lowered MDA values, the overall photon emission increased in *O. melanopus* infested leaves. However, the specific nature of the formed ROS will be a consequent step on the path of elucidating the concrete signaling pathways of *O. melanopus* infestation, some of which are independent of the direct lipid oxidation processes that the decrease of MDA level indicated along with the increase of overall biophoton emission signals. As a consequence of the investigations, it was confirmed that DF and UPE are both suitable for non-invasive way of biotic stress detection, triggered by *O. melanopus*. We identified that the antioxidant and lipid-oxidation–related physiological reactions were reflected in the dynamics of non-invasive biophoton emissions: both in the decay of DF and the consequent differences in UPE signals. Our research further supported that the non-invasive approach of stress assessment may complete and detail the traditional stress indicators, leading to a more precise estimation of the outcome of biotic stressors, even on a large scale.

Moreover, the physiological response of the damaged target organisms triggered by various stressors can be objectively judged by the application of this non-destructive imaging, which at the same time creates further research opportunities for the cognition of the inner stress advancement in the later vegetation stage of the same living plant material. In additional future perspectives, these non-destructive methods should play an increasingly determinative role in the research of plant-arthropod interaction to the more perfect discovery of hidden biological progressions. Eventually, the results originating from these methods can unequivocally contribute to both the development of integrated plant protection and the realization of reasonable pesticide utilization.

## Data Availability Statement

The raw data supporting the conclusions of this article will be made available by the authors, without undue reservation.

## Author Contributions

SK and IJ designed the methodology and wrote the manuscript. SK collected the research materials and set the experimental insect–host samples. IJ, KS-T, and HL performed the laboratory experiments, served the research, and performed the data evaluation. All authors read and approved the manuscript.

## Conflict of Interest

The authors declare that the research was conducted in the absence of any commercial or financial relationships that could be construed as a potential conflict of interest.

## Publisher’s Note

All claims expressed in this article are solely those of the authors and do not necessarily represent those of their affiliated organizations, or those of the publisher, the editors and the reviewers. Any product that may be evaluated in this article, or claim that may be made by its manufacturer, is not guaranteed or endorsed by the publisher.
