# Supplementary material for: Physiological Responses Manifested by Some Conventional Stress Parameters and Biophoton Emission in Winter Wheat as a Consequence of Cereal Leaf Beetle Infestation
Source: Front Plant Sci. 2022 Jul 6;13:839855. doi: 10.3389/fpls.2022.839855 (PMC9298668; doi:10.3389/fpls.2022.839855)
Supplement: Supplementary file 1 [file Data_Sheet_1.docx]

# #1 Supplementary material

# Statistical outputs of homogenous distribution and significant differences of the examined stress indices

**TABLE 1.** Statistical analysis of the examined stress parameters in damaged wheat caused by *O. melanopus:* Shapiro-Wilks normality test of fresh/dry weight ratio of chlorophyll content estimation (SPAD), antioxidant capacity (FRAP), lipid oxidation (MDA), delayed fluorescence (DF) and ultra-weak-photon emission (UPE). In the case of FRAP, the different distribution indicates the application of Flinger-Killeen test for not-normal distribution.

| **Shapiro-Wilks test** | | | |
| --- | --- | --- | --- |
| H_null: normal |  | p > 0.05 |  |
| H_alternative: not-normal |  | p < 0.05 |  |
|  | **p-value** | **Distribution** | **Applicable test for homogenity of variances** |
| **MDA control** | 0.2485 | Normal | Bartlett test |
| **MDA *O. melanopu*s infested** | 0.5903 | Normal | Bartlett test |
| **FRAP control** | 2.273*10^-3^ | Not-normal | Flinger-Killeen test |
| **FRAP *O. melanopus* infested** | 0.2322 | Normal | Flinger-Killeen test |
| **SPAD control** | 5.044*10^-5^ | Not-normal | Flinger-Killeen test |
| **SPAD *O. melanopus* infested** | 2,37*10^-5^ | Not-normal | Flinger-Killeen test |
| **UPE control** | 0.954 | Normal | Bartlett |
| **UPE *O. melanopus* infested** | 0.7361 | Normal | Bartlett |
| **DF control** | 1.17*10^-11^ | Not-normal | Flinger-Killeen test |
| **DF *O. melanopus* infested** | 2.52*10^-12^ | Not-normal | Flinger-Killeen test |

**TABLE 2.** Statistical analysis of the examined stress parameters in damaged wheat caused by *O. melanopus:* Bartlett test for normally distributed data and Flinger-Killeen test for not not-normally distributed data (TABLE 1.) of fresh/dry weight ratio of chlorophyll content estimation (SPAD), antioxidant capacity (FRAP), lipid oxidation (MDA), delayed fluorescence (DF) and ultra-weak-photon emission (UPE).

|  | **p-value** | **Results of homoscedasticity** |
| --- | --- | --- |
| **Lipid oxidation (MDA)** | 2.472×10^-7^ | There is significant difference between variances |
| **Antioxidant calpacity (FRAP)** | 1.076×10^-6^ | There is significant difference between variances |
| **Chlorophyll content estimation (SPAD)** | 2.2×10^-16^ | There is significant difference between variances |
| **Ultra-weak photon emission (UPE)** | 5.29×10^-14^ | There is significant difference between variances |
| **Delayed fluorescence (DF)** | 6.211×10^-3^ | There is significant difference between variances |

**TABLE 3.** Statistical analysis of the examined stress parameters in damaged wheat caused by *O. melanopus:* ANOVA analysis of fresh/dry weight ratio and Wilcoxon tests of chlorophyll content estimation (SPAD), antioxidant capacity (FRAP), lipid oxidation (MDA), delayed fluorescence (DF) and ultra-weak-photon emission (UPE)

| summary | Results of ANOVA for fresh/dry weight ratio | | | | | | |
| --- | --- | --- | --- | --- | --- | --- | --- |
| *groups* | *count* | *sum* | | *average* | *variance* |  |  |
| control | 5 | 45.318 | | 9.063 | 0.017 |  |  |
| *O. melanous* infested | 5 | 60.354 | | 12.071 | 0.106 |  |  |
| analysis of variance | | | | | | | |
| *source of variation* | *SS* | *df* | | *MS* | *F* | *p-value* | *F crit.* |
| between groups | 22.608 | 1 | | 22.608 | 384.891 | 5.8x10^-8^ | 5.317 |
| within groups | 0.495 | 8 | | 0.061 |  |  |  |
| total | 23.104 | 9 | |  |  |  |  |
|  | *p-value* | | Results of Wilcoxon test | | | | |
| MDA | 1.472×10^-7^ | | There are significant differences between groups | | | | |
| FRAP | 3.383×10^-6^ | |  |  |  |  |  |
| SPAD | 2.200×10^-16^ | |  |  |  |  |  |
| UPE | 2.200×10^-17^ | |  |  |  |  |  |
| DF | 1.520×10^-3^ | |  |  |  |  |  |
